# Guanfacine and HCN channels: bridging neuroinflammation and prefrontal cortex function in autism spectrum disorder

**DOI:** 10.3389/fnmol.2026.1915506

**Published:** 2026-09-09

**Authors:** Kazuhiko Yamamuro, Takahira Yamauchi, Manabu Makinodan

**Affiliations:** 1 Center for Health Control, Nara Medical University, Kashihara, Nara, Japan; 2 Department of Psychiatry, Nara Medical University, Kashihara, Nara, Japan; 3 Department of Neuropsychiatry, Kumamoto University, Kumamoto, Japan

**Keywords:** autism spectrum disorder, guanfacine, HCN channels, microglia, neuroinflammation, working memory, α2A-adrenoceptor

## Abstract

**Background:**

Autism spectrum disorder (ASD) is a neurodevelopmental condition characterized by social communication differences and restricted, repetitive behaviors. Convergent evidence implicates prefrontal cortex (PFC) circuit dysregulation and chronic neuroimmune activation in ASD. Despite increasing off-label use of guanfacine in children and adolescents with autism, its molecular and immunological rationale remains incompletely synthesized.

**Objective:**

To synthesize current evidence regarding the molecular, neurophysiological, and immunological mechanisms through which guanfacine may influence ASD-related neural and immune dysfunction.

**Methods:**

In this narrative review, we integrated evidence from pharmacology, systems neuroscience, immunology, and clinical studies to examine two converging mechanisms by which guanfacine may act in ASD and related conditions.

**Results:**

Guanfacine suppresses cAMP signaling through α2A-adrenoceptor activation, producing dual neuronal and immune effects. In PFC pyramidal neurons, reduced cAMP signaling promotes closure of hyperpolarization-activated cyclic nucleotide-gated (HCN) channels, strengthening network firing that supports working memory, attention, and emotion regulation. In parallel, activation of α2A-adrenoceptors on microglia and macrophages reduces production of pro-inflammatory cytokines, including tumor necrosis factor-α, interleukin-1β, and interleukin-6, while promoting anti-inflammatory phenotypes through nuclear factor kappa B suppression and peroxisome proliferator-activated receptor gamma activation. Evidence from human studies and ASD models indicates that α2A-adrenoceptor signaling, HCN channel function, and microglial reactivity are altered in autism and converge on synaptic refinement, dendritic spine stability, and PFC-dependent behavior. We further review clinical evidence for guanfacine in individuals with autism and in related conditions, including attention-deficit/hyperactivity disorder, post-traumatic stress disorder, traumatic brain injury, post-COVID cognitive impairment, delirium, and age-related cognitive decline.

**Conclusion:**

Collectively, the available evidence supports a mechanistic framework linking guanfacine, HCN channel modulation, and neuroimmune regulation, thereby bridging neuroinflammation and PFC function in ASD. The broader α2A–HCN–microglia axis may represent a promising therapeutic target for PFC- and neuroimmune-related features of ASD; however, adequately powered autism-specific randomized trials and biomarker-informed stratification strategies are needed to establish clinical efficacy and validate this framework.

## 1 Introduction

The prefrontal cortex (PFC) is central to goal-directed behavior, working memory, and executive control (Arnsten, 2020; Fesharaki-Zadeh et al., 2023a; Goldman-Rakic, 1995; Miller and Cohen, 2001). Pioneering electrophysiological work established persistent neuronal firing in PFC circuits as a neural substrate of working memory (Funahashi, 2006), and subsequent studies showed that catecholaminergic modulation regulates these recurrent networks (Arnsten and Goldman-Rakic, 1985; Vijayraghavan et al., 2007). Dopaminergic and noradrenergic inputs jointly influence spatial working memory performance (Sawaguchi and Goldman-Rakic, 1991; Williams and Goldman-Rakic, 1995), while computational models have emphasized the importance of recurrent connectivity in stabilizing persistent activity (Constantinidis and Wang, 2004; Durstewitz et al., 2000). These findings highlight the vulnerability of PFC-dependent cognition to neuromodulatory and molecular perturbations, particularly in neurodevelopmental conditions including autism spectrum disorder (ASD).

ASD is increasingly understood as a condition wherein atypical PFC connectivity, altered noradrenergic regulation, and chronic low-grade neuroimmune activation co-occur and interact (Arnsten et al., 2023; Kana et al., 2014; Masi et al., 2015). Functional and structural neuroimaging studies in individuals with autism have identified atypical frontal connectivity—encompassing both structural (diffusion and morphometric) and functional (resting-state and task-based) measures—and altered prefrontal recruitment during executive tasks (Just et al., 2007; Kana et al., 2014; Uddin et al., 2013). Postmortem and immune-profiling studies likewise document microglial activation, elevated brain and peripheral cytokine levels (notably interleukin (IL)-1β, IL-6, and tumor necrosis factor (TNF)-α), and altered complement signaling in a substantial subset of individuals with autism (Ashwood et al., 2011; Druart and Le Magueresse, 2019; Masi et al., 2015; Morgan et al., 2010; Vargas et al., 2005). These convergent observations raise the possibility that interventions capable of simultaneously stabilizing PFC network firing and dampening neuroimmune activation could address a mechanistically meaningful axis of ASD pathophysiology, rather than only its symptomatic surface. Although the immune changes described above are frequently described in relation to the PFC, they are not confined to it. For example, microglial activation and cytokine elevations in autism have been reported across the cerebral cortex, cerebellum, hippocampus, and striatum, as well as in cerebrospinal fluid and peripheral blood, indicating a brain-wide and somewhat systemic immune phenotype (Suzuki et al., 2013; Tetreault et al., 2012; Vargas et al., 2005). We nevertheless focused on the PFC not because the immune changes are anatomically selective, but because the PFC is disproportionately vulnerable to their downstream consequences: its recurrent networks depend on a narrow optimum of cAMP–HCN signaling, its dendritic spines are unusually sensitive to inflammatory and stress mediators, and quantitative postmortem work has reported the most consistent increases in microglial density and activation in the dorsolateral PFC (Arnsten et al., 2023; Morgan et al., 2010). Striatal, hippocampal, and sensory-cortical circuits are also affected in autism and plausibly contribute to repetitive behavior, associative memory, and sensory hyperreactivity; the framework developed here should therefore be read as PFC-weighted rather than PFC-exclusive.

Chronic stress disrupts PFC network integrity and is highly relevant to autism, given the elevated stress reactivity and high rates of anxiety reported in autistic populations. Prolonged stress exposure induces structural remodeling of PFC pyramidal neurons (Liston et al., 2006; Radley et al., 2004), impairing cognitive flexibility and executive function (Holmes and Wellman, 2009; McEwen and Morrison, 2013). Stress-related elevations in catecholamines destabilize recurrent network activity (Arnsten, 2009; Roozendaal et al., 2009), and glucocorticoid signaling further contributes to impaired executive control (Joëls and Baram, 2009; Shansky and Lipps, 2013). These cascades result in long-lasting alterations in cortical behavioral regulation (Arnsten, 2015; McKlveen et al., 2016). Chronic stress also activates neuroimmune pathways that further compromise PFC circuit stability. Neuroinflammatory responses involve coordinated activation of microglia and peripheral immune cells (Perry et al., 2010; Ransohoff and Brown, 2012). Cytokine signaling modulates synaptic plasticity, neuronal survival, and network excitability (Block et al., 2007; Cunningham et al., 2009); and microglial activation is increasingly recognized as a regulator of cognitive trajectories (Glass et al., 2010; Hanisch and Kettenmann, 2007). Immune mediators alter both structural connectivity—through effects on dendritic spine turnover and synaptic pruning—and functional connectivity as measured via neuroimaging, and thereby disrupt higher-order cognition (Harrison et al., 2009; Kreutzberg, 1996; Marsland et al., 2008). Together, these findings position neuroimmune interactions as a critical interface, particularly in ASD, through which stress and developmental signals impair PFC-dependent cognition.

Dysfunction of PFC networks underlies cognitive deficits in numerous neuropsychiatric and neurodevelopmental conditions, often exacerbated by stress and inflammation (Arnsten et al., 2023; Tan et al., 2023). Growing evidence indicates that neuroimmune interactions impair PFC structural and functional connectivity and cognition, as peripheral and central inflammation disrupts synaptic function and network dynamics (Lynch, 2010; Tan et al., 2023). Inflammatory cytokines released by activated microglia or infiltrating macrophages weaken synaptic plasticity and memory processes (Cibelli et al., 2010; Lynch, 2010). Consequently, interventions that reduce neuroinflammation can preserve or restore cognitive function (Fesharaki-Zadeh et al., 2023b; Skelly et al., 2018). Inflammatory cytokines disrupt dendritic spine stability in prefrontal neurons (Haroon and Miller, 2016; Miller et al., 2009); peripheral inflammation alters frontoparietal connectivity in human neuroimaging (Harrison et al., 2009; Marsland et al., 2008); and elevated IL-6 correlates with reduced working memory performance (Felger et al., 2016). These findings suggest that systemic immune activation directly influences higher-order cognitive processes (McAfoose and Baune, 2009; Yirmiya et al., 2015), a pathway clearly relevant to the cognitive and behavioral features of ASD.

**FIGURE 1:**
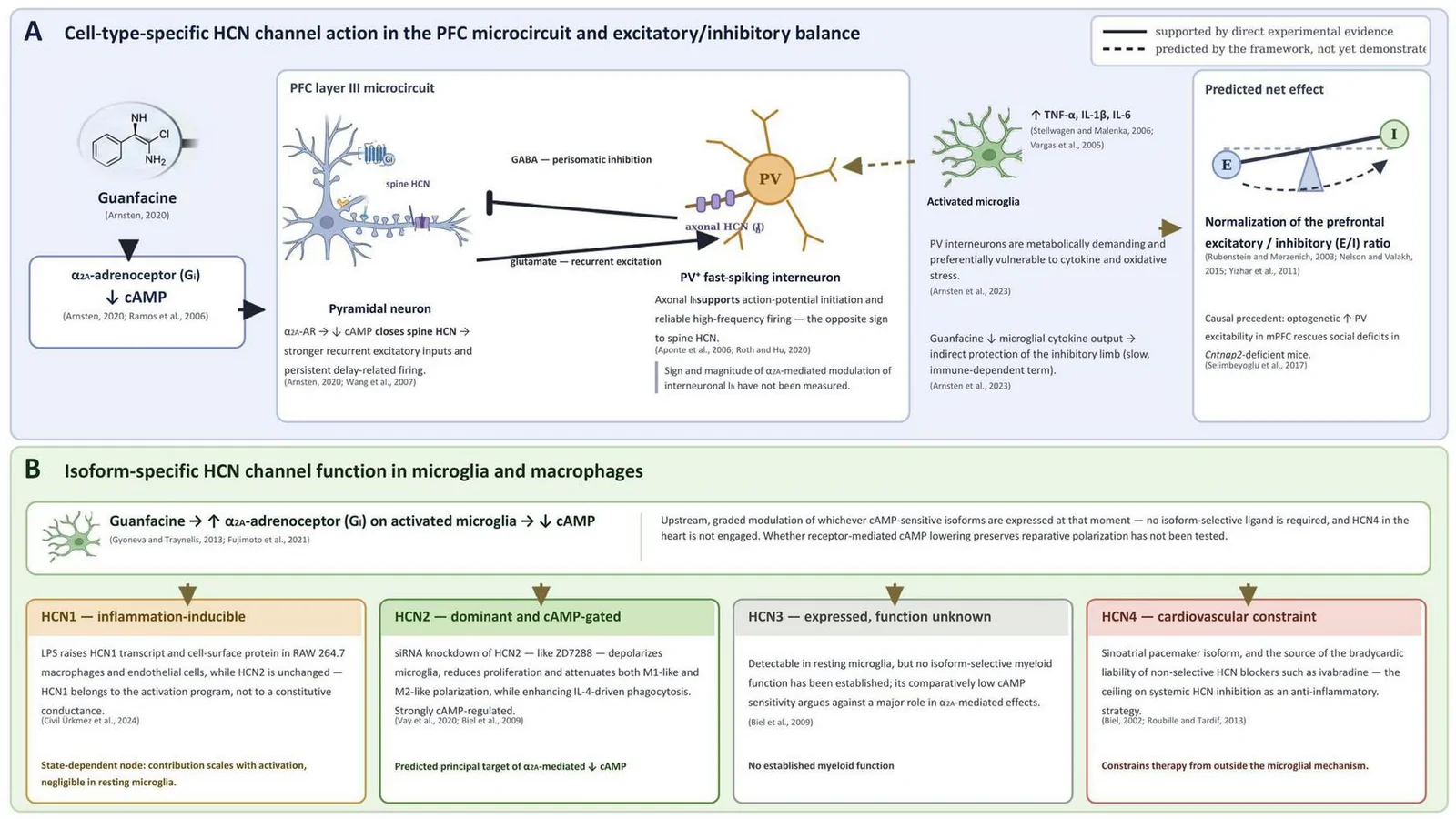
Cell-type-specific and isoform-specific HCN channel function in the prefrontal cortical microcircuit and in myeloid cells. **(A)** Cell-type-specific action of HCN channels in the prefrontal cortical microcircuit. In pyramidal neurons (left), α2A-adrenoceptor (α2A-AR) stimulation by guanfacine lowers cyclic adenosine monophosphate (cAMP) and closes HCN channels on dendritic spines, strengthening recurrent excitatory connections and delay-related firing. In parvalbumin (PV)-expressing fast-spiking interneurons (right), the hyperpolarization-activated current (I_h) is concentrated in the axon and supports reliable high-frequency firing, so HCN channels act on the inhibitory limb of the microcircuit with opposite sign. Neuroinflammation, mediated by activated microglia and pro-inflammatory cytokines, preferentially compromises metabolically demanding PV interneurons. The predicted net effect of α2A stimulation is normalization of the excitatory/inhibitory (E/I) ratio, together with indirect protection of interneurons through reduced microglial cytokine output. In **(A)**, solid lines denote relationships supported by direct experimental evidence and dashed lines denote relationships that are predicted by the framework but not yet demonstrated, which are discussed as such in the text. **(B)** Isoform-specific HCN channel function in microglia and macrophages. HCN1 is upregulated by lipopolysaccharide (LPS) and forms part of the inflammatory activation program; HCN2 is constitutively expressed, is unchanged by LPS stimulation, is strongly cAMP-regulated, and drives proliferation and polarization toward both M1-like and M2-like states; HCN3 has no established myeloid function; HCN4 is the sinoatrial pacemaker isoform and is the source of the bradycardic liability of non-selective HCN blockers. The proposed framework places guanfacine upstream of all four isoforms, lowering cAMP through Gi-coupled α2A-adrenoceptors and thereby providing graded modulation without requiring isoform-selective ligands. In **(B)**, the upstream limb (guanfacine →α2A-adrenoceptor on activated microglia →↓cAMP) is annotated in the figure as untested for guanfacine itself, and whether receptor-mediated lowering of cAMP preserves reparative polarization has not been examined. α2A-AR, α2A-adrenoceptor; cAMP, cyclic adenosine monophosphate; E/I, excitatory/inhibitory; GABA, gamma-aminobutyric acid; HCN, hyperpolarization-activated cyclic nucleotide-gated; I_h, hyperpolarization-activated current; IL, interleukin; LPS, lipopolysaccharide; PFC, prefrontal cortex; PV, parvalbumin; TNF-α, tumor necrosis factor alpha.

The molecular mechanisms linking neuromodulatory signaling, ion channel regulation, and neuroimmune interactions within PFC circuits remain incompletely understood, particularly in the context of ASD. Here, we review how guanfacine—a selective α2A-adrenergic receptor agonist used clinically for PFC-related disorders, including in autism—interacts with hyperpolarization-activated cyclic nucleotide-gated (HCN) channels and with microglia/macrophages to modulate PFC circuitry and neuroinflammation. We synthesize findings across pharmacology, neuroscience, and immunology, highlighting that guanfacine’s pro-cognitive effects involve closure of HCN channels in PFC neurons (Arnsten, 2020; Wang et al., 2007) and putative immunomodulatory actions on microglia and macrophages, for which direct guanfacine-specific evidence remains limited (Arnsten, 2020). We also discuss the implications for ASD, where co-occurring inattention, irritability, hyperarousal, and sensory dysregulation are commonly treated symptomatically and where the mechanistic rationale for α2A-targeted intervention has not been systematically articulated. We organize the evidence around two reciprocal signaling axes that share HCN channels as a common molecular node: a guanfacine–HCN–neuron axis, through which α2A-mediated lowering of cAMP closes HCN channels and shapes prefrontal excitatory/inhibitory balance, and a guanfacine–HCN–neuroinflammation axis, through which the same receptor and channel machinery in microglia and macrophages may constrain inflammatory activation (Figure 1).

## 2 Guanfacine and clonidine: α2A-adrenoceptor pharmacology, HCN channel modulation, and clinical efficacy in ASD

Role of noradrenergic modulation has long been implicated in executive processes, particularly through prefrontal cortical networks (Arnsten, 2011; Robbins and Arnsten, 2009). Animal and human studies have reported that pharmacological manipulation of α2-adrenoceptors enhances cognitive flexibility (Chamberlain et al., 2009; Coull et al., 2011), with effects particularly pronounced under stress or cognitive fatigue (Berridge and Waterhouse, 2003; Spencer et al., 2015). These findings support the therapeutic potential of α2A agonists across multiple neuropsychiatric conditions, including ASD (Aston-Jones and Cohen, 2005; Sara, 2009).

Guanfacine, an α2A-adrenergic receptor agonist originally developed as an antihypertensive, is now widely used to treat attention-deficit/hyperactivity disorder (ADHD) and other PFC-mediated cognitive disorders (Arnsten, 2020). It was approved for pediatric ADHD in 2009 following evidence that it enhances PFC function. Multiple randomized trials have shown that guanfacine extended-release (GXR) significantly improves ADHD symptoms and is well-tolerated in children and adolescents (Arnsten, 2020; Iwanami et al., 2020). These benefits extend to adults, with a phase 3 trial in Japan reporting a greater reduction in ADHD rating scores in response to GXR than to placebo (Iwanami et al., 2020).

Guanfacine has long been used off-label in autism, and a placebo-controlled trial reported clinically meaningful reductions in hyperactivity, supporting its use for ASD-associated hyperactivity and impulsivity (Scahill et al., 2015). Guanfacine is also prescribed off-label for Tourette’s syndrome, traumatic brain injury (TBI), and post-traumatic stress disorder (PTSD) (Millichap, 2011). Small studies suggest that guanfacine improves executive function and impulse control across these conditions, although outcomes vary. An open-label trial reported reduced PTSD-related hyperarousal in children receiving GXR (Arnsten, 2020; Connor et al., 2013), whereas a controlled trial in veterans with chronic PTSD found no significant benefit for core PTSD symptoms (Arnsten, 2020; Neylan et al., 2006), suggesting efficacy may depend on population and cognitive domain.

A randomized controlled trial in older adults (aged ≥ 75 years) found no significant improvement in PFC-dependent task performance with guanfacine versus placebo (Barcelos et al., 2018), suggesting greater efficacy with earlier intervention before age-related synaptic changes become irreversible (Arnsten, 2020). The translational success of guanfacine across species and disorders, together with its favorable side-effect profile in pediatric populations, including children with autism, highlights the importance of its actions on PFC circuitry (Table 1). The subsequent sections elucidate the mechanisms underlying the effects of guanfacine, focusing on cyclic adenosine monophosphate (cAMP)-dependent HCN channel regulation and emerging evidence for neuroimmune modulation.

**TABLE 1:** Selected clinical studies of guanfacine in neuropsychiatric and cognitive conditions relevant to autism spectrum disorder.

Study (year)	Indication	Population/design	Key findings	References
Sallee et al. (2009)	ADHD	Children/adolescents (6–17 y); RCT, dose-ranging	Dose-dependent improvement in ADHD-RS-IV; led to FDA approval in 2009	(Sallee et al., 2009)
Iwanami et al. (2020)	ADHD (adults)	Adult Japanese patients; Phase 3 RCT	Significant reduction in ADHD-RS vs. placebo in adults	(Iwanami et al., 2020)
Connor et al. (2013)	PTSD/trauma symptoms	Children/adolescents; open-label, 8 weeks	Reduced hyperarousal, re-experiencing, avoidance symptoms	(Connor et al., 2013)
Neylan et al. (2006)	Chronic PTSD	Veterans; RCT, placebo-controlled	No significant benefit on core PTSD symptoms	(Neylan et al., 2006)
Barcelos et al. (2018)	Age-related cognitive decline	Healthy older adults ( ≥ 75 y); RCT, 12 weeks	Did not improve PFC executive function vs. placebo	(Barcelos et al., 2018)
McAllister et al. (2011)	Mild TBI	Adults with mild TBI; single-dose challenge	Altered PFC BOLD response; modest working memory improvement	(McAllister et al., 2011)
Singh-Curry et al. (2011)	Autoimmune encephalitis (ADEM)	Single case report	Improvement in attention and function (single case; no quantitative outcome measures reported)	(Singh-Curry et al., 2011)
Fesharaki-Zadeh et al. (2023b)	Long COVID cognitive deficits	Open-label pilot with NAC (12 patients)	Improved working memory and executive function in 8/12 patients	(Fesharaki-Zadeh et al., 2023b)
Fesharaki-Zadeh et al. (2025)	Severe TBI	Case series with guanfacine + NAC + donepezil	Improved working memory and impulsivity	(Fesharaki-Zadeh et al., 2025)

The included studies were selected to illustrate clinical applications of guanfacine in conditions involving prefrontal cortical dysfunction and are not derived from a systematic review. Study populations, diagnoses, treatment durations, guanfacine formulations, and outcome measures differed substantially; therefore, findings should not be compared directly across studies. Most studies were not conducted in individuals with autism spectrum disorder and do not establish efficacy for core autism features. In the Long COVID and severe TBI reports, guanfacine was administered with NAC or with NAC and donepezil, respectively; consequently, the contribution of guanfacine cannot be isolated. The open-label studies and case reports lacked placebo control, and no inflammatory biomarkers were measured; thus, they do not provide direct clinical evidence of a guanfacine-specific immunomodulatory effect. ADEM, acute disseminated encephalomyelitis; ADHD, attention-deficit/hyperactivity disorder; ADHD-RS-IV, Attention-Deficit/Hyperactivity Disorder Rating Scale IV; BOLD, blood-oxygen-level-dependent; FDA, US Food and Drug Administration; NAC, N-acetylcysteine; PFC, prefrontal cortex; PTSD, post-traumatic stress disorder; RCT, randomized controlled trial; TBI, traumatic brain injury.

Within this pharmacological framework, guanfacine must be distinguished from clonidine, because the two α2 agonists diverge in receptor selectivity, and these differences propagate to HCN channel modulation, immunomodulatory capacity, and clinical utility in ASD. Clonidine is a relatively non-selective α2 agonist with comparable affinity for the α2A, α2B, and α2C subtypes and appreciable activity at imidazoline I1 receptors, whereas guanfacine is preferentially selective for α2A over α2B and α2C and lacks meaningful imidazoline activity (Arnsten, 2020; Arnsten et al., 1988). Because the pro-cognitive action in the PFC is mediated by postsynaptic α2A receptors, whereas sedation and hypotension arise largely from α2A autoreceptors in the locus coeruleus together with α2B-mediated vascular and central actions, guanfacine attains cognitive enhancement at doses both less sedating and hypotensive. In aged monkeys, guanfacine improved working memory with markedly fewer sedative and hypotensive effects than clonidine, which provided the original evidence for functionally distinct α2 receptor subtypes (Arnsten et al., 1988).

With respect to HCN channel modulation, the postsynaptic cAMP–HCN mechanism has been characterized almost exclusively with guanfacine and α2A-selective tools. Iontophoretic guanfacine increases delay-related firing in the dorsolateral PFC, an effect reversed by cAMP analogs and mimicked by HCN channel blockade (Wang et al., 2007). Clonidine, acting at the same Gi-coupled α2A receptor, would in principle close spine HCN channels by the same route; however, its α2A-autoreceptor-driven reduction in noradrenergic tone and its α2B/α2C actions produce a competing, predominantly sedative profile, and comparative electrophysiological studies of spine HCN modulation by the two agents have not been performed. Direct comparative data on HCN channel effects are therefore unavailable, and the apparent advantage of guanfacine at the level of PFC physiology is best attributed to receptor selectivity and site of action rather than to a distinct channel mechanism.

Although direct comparative data are absent, the expected divergence can be inferred, thereby leading to the identification what needs to be measured. At the level of the individual dendritic spine, clonidine and guanfacine should be qualitatively similar, since both are agonists at the same Gi-coupled α2A receptor, and the transduction step—inhibition of adenylyl cyclase with consequent closure of HCN channels—is receptor-specific rather than ligand-specific; if anything, the higher intrinsic efficacy of clonidine at α2A would predict equal or greater spine HCN closure at matched receptor occupancy. The divergence is expected at the network level, and it arises from two additional actions that guanfacine largely lacks. First, potent stimulation by clonidine of somatodendritic α2A autoreceptors in the locus coeruleus reduces the noradrenergic drive that supplies the transmitter required to occupy postsynaptic α2A receptors in the PFC, so the presynaptic and postsynaptic actions are partially self-canceling and the dose–response relationship for cognitive benefit should be correspondingly narrow. Second, α2B-mediated sedation and hypotension degrade performance on any attention-dependent task independently of channel state. The resulting prediction is specific and testable: at matched α2A occupancy *in vitro*, clonidine and guanfacine should produce comparable reductions in dendritic I_h, whereas *in vivo* clonidine should exhibit a lower and left-shifted peak of the inverted-U dose–response curve for delay-related firing and working memory. Iontophoretic application of the two agents within the same recording sessions, combined with paired slice measurements of dendritic I_h, would settle the question; this experiment is feasible with existing techniques but has simply not been performed.

Immunomodulatory data are similarly asymmetric. Anti-inflammatory α2 effects—suppression of TNF-α, IL-6, and IL-1β, elevation of IL-10, and PPAR-γ engagement—have been demonstrated most robustly for dexmedetomidine, an α2A-preferring agonist, and to a lesser extent for clonidine in perioperative and sepsis models, whereas direct guanfacine-specific myeloid evidence remains limited; the available rationale is derived largely from studies of norepinephrine, dexmedetomidine, and other α2-adrenoceptor agonists (Fujimoto et al., 2021; Han et al., 2022; Nayak et al., 2024). Because activated microglia selectively upregulate α2A-adrenoceptors (Gyoneva and Traynelis, 2013), α2A selectivity is predicted to be advantageous for targeting reactive microglia while sparing β2-mediated homeostatic signaling in resting cells. Head-to-head immunological comparisons of guanfacine and clonidine have nonetheless not been reported, and this prediction remains untested.

Clinically, the evidence base in autism favors guanfacine. Extended-release guanfacine reduced hyperactivity, impulsiveness, and distractibility in a placebo-controlled trial in children with ASD and in its secondary analysis, with a large effect size on the primary outcome (Cohen’s *d* of approximately 1.7 on the Aberrant Behavior Checklist hyperactivity subscale, corresponding to a reduction of approximately 44% from baseline versus approximately 13% with placebo, and a rate of much or very much improvement on the Clinical Global Impression–Improvement scale of approximately 50% vs. 9.4%), which exceeds the effect sizes typically reported for stimulants in this population; benefits were also observed on secondary measures of inattention and oppositional behavior (Politte et al., 2018; Scahill et al., 2015). These two reports describe a single randomized trial and its secondary analysis rather than independent replications. The gains were moreover confined to behavioral domains, since the same trial found no between-group difference on cognitive battery outcomes; improvement in hyperactivity and impulsivity in ASD should therefore not be equated with cognitive benefit. Clonidine trials in autism are older, smaller, and shorter in duration; although both a double-blind transdermal study and an oral crossover study reported reductions in hyperactivity and irritability, sedation was frequently dose-limiting and tolerance was described (Fankhauser et al., 1992; Jaselskis et al., 1992). No randomized head-to-head comparison of the two agents exists in ASD. On the available evidence, guanfacine is preferable when daytime control of hyperactivity, impulsivity, and attentional symptoms is the therapeutic goal, whereas the greater sedative potency of clonidine may be exploited when sleep-onset insomnia or acute hyperarousal is the primary target.

## 3 HCN channels: isoforms, cellular distribution, and functional phenotypes in neurons and myeloid cells

HCN channels are hyperpolarization-activated, cAMP modulated cation channels (Biel et al., 2009). Four HCN subunits (HCN1–4) assemble as homo- or heterotetramers that carry the hyperpolarization-activated I_h current. In neurons, I_h regulates resting membrane potential, dendritic excitability, and rhythmic oscillations. HCN channels are widely expressed in the brain, particularly HCN1/2 in the cortex and hippocampus and in the heart where HCN4 drives pacemaker activity (Biel et al., 2002; Civil Ürkmez et al., 2024). They are also expressed in immune cells, including microglia and macrophages (Table 2). HCN channel activity is sensitive to intracellular cAMP: cAMP binding induces HCN channels to open at less negative potentials, effectively increasing excitability (Biel et al., 2009). In cortical pyramidal neurons, dendritic HCN channels act as a “leak” conductance that dampens synaptic inputs and repetitive firing (Arnsten, 2020), and thus excessive I_h weakens synaptic integration.

**TABLE 2:** Cell type-specific expression and functions of HCN channel isoforms.

Cell type	Predominant subunit(s)	Functional role	Modulation/inhibition effect	References
PFC pyramidal neurons	HCN1, HCN2 (dendritic)	Dampen distal synaptic inputs; cAMP-gated weakening of recurrent firing	Closure (by α2A agonists) strengthens working memory firing	(Arnsten, 2020; Kettenmann et al., 2011; Wang et al., 2007)
Hippocampal neurons	HCN1, HCN2	Set resting potential; pace theta oscillations; influence memory consolidation	Blockade (e.g., ZD7288) improves spatial memory after ischemia	(Robinson and Siegelbaum, 2003; Roubille and Tardif, 2013)
Cardiac pacemaker cells (SAN)	HCN4	Generate spontaneous diastolic depolarization (*I*_*f*_ current)	Blockade (ivabradine) slows heart rate	(Civil Ürkmez et al., 2024; Pinares-Garcia et al., 2023)
Microglia	All four subunits (HCN1–4)	Maintain hyperpolarized state; influence Ca^2+^ signaling and activation tone	Blockade reduces proliferation and M1-like/M2-like polarization; enhances phagocytosis under M2 conditions	(Biel et al., 2002)
Peripheral macrophages	HCN1 (predominant after LPS)	Expression upregulated by LPS; role in inflammatory response	HCN1 may serve as marker/regulator of macrophage activation	(Roubille and Tardif, 2013)
Endothelial cells	HCN1 (induced by LPS)	Possibly involved in inflammation-related signaling	Function under investigation	(Roubille and Tardif, 2013)

HCN isoform expression and function vary according to species, cell type, developmental stage, and activation state. Evidence for HCN1–HCN4 expression and function in microglia is derived primarily from cultured rodent cells, whereas LPS-induced HCN1 expression in macrophages and endothelial cells has been demonstrated mainly in cell-line models. No study has established isoform-specific HCN function in human microglia or macrophages *in vivo* or in autism spectrum disorder. ZD7288 is a non-isoform-selective HCN channel blocker with potential off-target effects. The terms M1-like and M2-like are used only to describe the experimental polarization conditions employed in the cited studies and do not imply fixed or mutually exclusive microglial states. cAMP, cyclic adenosine monophosphate; HCN, hyperpolarization-activated cyclic nucleotide-gated; I_f, funny current; LPS, lipopolysaccharide; M1/M2, M1-like/M2-like polarization states; PFC, prefrontal cortex; SAN, sinoatrial node.

HCN channel dysfunction is implicated in epilepsy, neuropathic pain, depression, and cognitive aging (He et al., 2025; Pinares-Garcia et al., 2023; Roubille and Tardif, 2013), and increasingly induced in neurodevelopmental conditions, including ASD. In mouse models of depression, stress-induced elevations of cAMP signaling in the PFC and hippocampus are associated with excessive HCN channel activity and behavioral deficits (Arnsten et al., 2023; Roubille and Tardif, 2013). Conversely, pharmacological inhibition or genetic knockdown of HCN channels induces antidepressant-like and pro-cognitive effects, including improved spatial memory in rodents following treatment with HCN blocker ZD7288 (Ku and Han, 2017; Roubille and Tardif, 2013). HCN channels have therefore been proposed as potential novel targets for antidepressants given their influence on neuronal excitability and plasticity (Ku and Han, 2017; Robinson and Siegelbaum, 2003; Roubille and Tardif, 2013). They also contribute to dendritic signal integration in pyramidal neurons (Lewis and Chetkovich, 2011; Shah, 2014) and altered HCN expression is linked to cognitive deficits in aging and stress models (Kazmierska-Grebowska et al., 2024; Nolan et al., 2007; Thuault et al., 2013), pharmacological inhibition of HCN channels improves synaptic efficacy in prefrontal circuits (Kim et al., 2012; Wang et al., 2011). These findings underscore HCN channel regulation as a critical node for executive function (Paspalas et al., 2013; Ramos and Arnsten, 2007).

**FIGURE 2:**
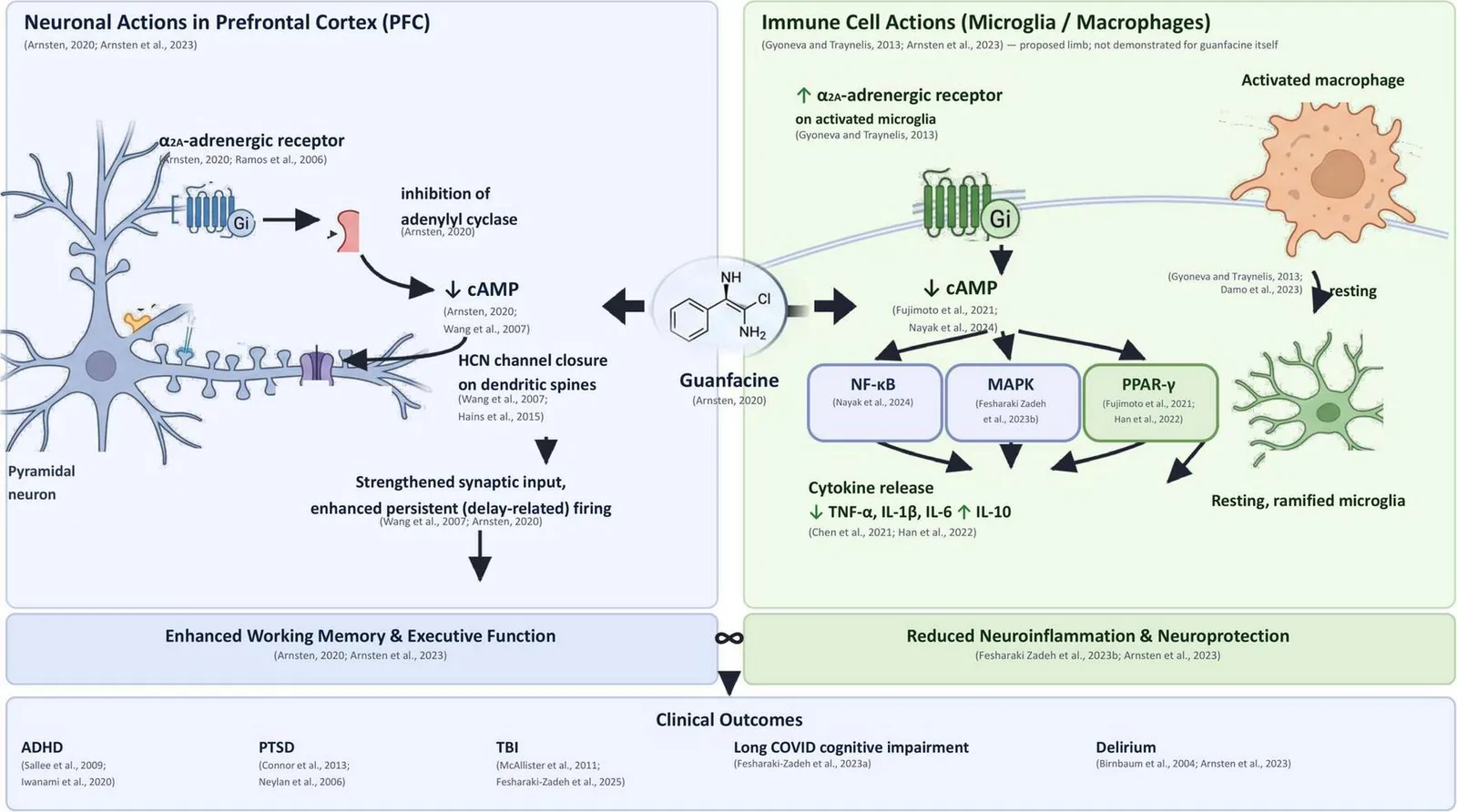
Neuronal and immune actions of guanfacine. Dual mechanisms of guanfacine in prefrontal cortex neurons and immune cells. Guanfacine stimulates α2A-adrenoceptors (α2A-AR) coupled to inhibitory Gi proteins in both pyramidal neurons of the prefrontal cortex (PFC; left panel) and activated microglia/macrophages (right panel). In PFC pyramidal neurons, α2A-AR activation reduces intracellular cyclic adenosine monophosphate (cAMP), leading to closure of hyperpolarization-activated cyclic nucleotide-gated (HCN) channels on dendritic spines. This strengthens synaptic inputs and promotes persistent delay-related neuronal firing, thereby enhancing working memory and executive function. In microglia and macrophages, the same Gi-coupled reduction in cAMP is proposed to suppress pro-inflammatory NF-κB and MAPK signaling pathways while activating PPAR-γ. These downstream changes would be expected to decrease pro-inflammatory cytokines (TNF-α, IL-1β, IL-6) and elevate IL-10, favoring a shift from an activated toward a resting, ramified phenotype. This immune limb of the schematic is inferred from the pharmacology of α2A-adrenoceptor agonism and from studies of norepinephrine and of other α2 agonists; it has not been demonstrated for guanfacine directly and is therefore depicted as a proposed rather than an established mechanism. The convergence of strengthened prefrontal cortical function and attenuated neuroinflammation may underlie guanfacine’s translational potential for executive dysfunction and neuroinflammation-related cognitive symptoms across multiple neuropsychiatric conditions, including attention-deficit/hyperactivity disorder (ADHD; Sallee et al., 2009; Iwanami et al., 2020), post-traumatic stress disorder (PTSD), traumatic brain injury (TBI), Long COVID-associated cognitive impairment, and delirium. α2A-AR, α2A-adrenoceptor; cAMP, cyclic adenosine monophosphate; HCN, hyperpolarization-activated cyclic nucleotide-gated; IL, interleukin; MAPK, mitogen-activated protein kinase; NF-κB, nuclear factor kappa B; PFC, prefrontal cortex; PPAR-γ, peroxisome proliferator-activated receptor gamma; TNF-α, tumor necrosis factor alpha.

In summary, HCN channels are key regulators of neuronal firing and network stability (Figure 2A). Their unique biophysical profile—opening under cAMP-mediated hyperpolarization—positions them at the crossroads of electrical and biochemical signaling. This makes HCN channels critical mediators of neuromodulation in the PFC and suggests that peripheral signals (such as inflammatory mediators that alter cAMP) could indirectly affect neuronal HCN activity, with potential implications for ASD-associated cognitive features (Cortese et al., 2018).

The same channel family is not confined to neurons: HCN channels are also expressed in myeloid cells of the central nervous system and the periphery, and their isoform repertoire, cellular distribution, and functional phenotypes in these cells are considered next. Microglia (resident macrophages of the central nervous system) play key roles in synaptic refinement during development and in the adult brain (Colonna and Butovsky, 2017; Wolf et al., 2017). This process is implicated in ASD pathogenesis, where altered microglia–synapse interactions are a leading mechanistic hypothesis (Paolicelli et al., 2011; Schafer et al., 2012; Vargas et al., 2005). Activated microglia release cytokines that impair synaptic transmission (Stellwagen and Malenka, 2006; Viviani et al., 2003). Ion channel activity contributes to microglial activation states, and modulation of microglial signaling may influence cognitive and behavioral outcomes (Gyoneva and Traynelis, 2013; Wohleb et al., 2016).

Although classically studied in neurons and cardiomyocytes, HCN channels are also expressed in non-excitable cells, including macrophages and microglia. Recent research indicates that immune activation can upregulate HCN channel expression and modulate immune cell function (Pinares-Garcia et al., 2023). An *in vitro* inflammation model using macrophage (RAW 264.7) and endothelial (HUVEC) cell lines showed that bacterial lipopolysaccharide (LPS) significantly upregulated HCN1 gene and protein expression levels, whereas HCN2 expression remained unchanged. HCN1 protein expression on the cell membrane was also elevated following LPS stimulation, suggesting that HCN1 is the dominant HCN subtype in macrophages and may contribute to inflammatory responses. These findings indicate that upregulation of HCN1 expression could be part of the macrophage activation profile (Civil Ürkmez et al., 2024).

Microglia express all four HCN subunits under basal conditions (Biel et al., 2009). Vay et al. (2020) demonstrated the functional roles of these channels in microglial physiology. Using primary rat microglia, they showed that pharmacological blockade of HCN channels with ZD7288 caused depolarization and reduction in proliferation but enhanced survival of resting microglia. Inhibiting HCN (via ZD7288 or siRNA-mediated knockdown of HCN2) attenuated microglia activation toward both pro-inflammatory (M1-like) and anti-inflammatory (M2-like) phenotypes upon LPS or IL-4 stimulation. In essence, blocking HCN currents made microglia less reactive. Certain functions of microglia were however augmented. Notably, microglial phagocytic capacity under IL-4 (M2) conditions was increased by HCN blockade. These observations suggest that HCN channel activity contributes to the orchestration of microglial activation and motility. By maintaining a hyperpolarized membrane and influencing Ca^2+^ signaling, HCN channels may help drive microglia toward an activated, proliferative state. Consistent with this, another study reported that HCN inhibition bolstered microglial phagocytosis, potentially aiding clearance of debris during repair. The presence of HCN channels in macrophages and microglia adds an intriguing layer to neuroimmune communication. Although traditionally “non-excitable,” these immune cells maintain a membrane potential and express various ion channels that influence their behavior (e.g., Kv1.3, KCa3.1). HCN channels join this roster of ion channels regulating immune cell function. Inflammatory factors can induce HCN expression, and conversely, ion channel activity can affect immune signaling pathways. Microglial HCN blockade modulated intracellular Ca^2+^ transients and downstream activation cascades (Biel et al., 2009; Vay et al., 2020).

Because the four HCN isoforms differ in cAMP sensitivity, activation kinetics, and tissue distribution, their contributions to myeloid biology are not interchangeable; Table 3 compiles the isoform-resolved evidence currently available. HCN1 behaves as the inflammation-inducible isoform: LPS stimulation upregulates HCN1 transcript and surface protein in RAW 264.7 macrophages and in endothelial cells, whereas HCN2 expression is unchanged, indicating that HCN1 forms part of the activation program rather than a constitutive housekeeping conductance (Civil Ürkmez et al., 2024). HCN2 appears to be the functionally dominant isoform in microglia: siRNA-mediated knockdown of *HCN2*, like pharmacological blockade with ZD7288, depolarizes microglia, reduces proliferation, and attenuates polarization toward both pro-inflammatory M1-like and reparative M2-like states, while paradoxically enhancing IL-4-driven phagocytosis (Vay et al., 2020). HCN2 is also strongly modulated by cAMP in neurons—a property it shares with HCN4—which provides a plausible mechanistic link between Gi-coupled α2A signaling and myeloid HCN function (Biel et al., 2009). HCN3 and HCN4 are detectable in resting microglia, but no isoform-selective functional or disease role has been established for either; HCN4, the pacemaker isoform of the sinoatrial node, is chiefly relevant as the source of the bradycardic liability of non-selective HCN blockers, such as ivabradine, a constraint that limits systemic HCN inhibition as an anti-inflammatory strategy (Biel et al., 2002; Roubille and Tardif, 2013).

**TABLE 3:** Current evidence and proposed therapeutic implications of HCN channel isoforms in microglia and macrophages.

Isoform	Expression in microglia/ macrophages	Reported function	Disease and therapeutic implications	References
HCN1	Low at baseline; strongly upregulated by LPS in RAW 264.7 macrophages and endothelial cells (transcript and surface protein); present in resting microglia	Upregulated as part of the inflammatory activation program; its isoform-specific functional contribution in myeloid cells has not been established	Hypothesized activation-state-dependent target; whether HCN1-selective inhibition attenuates macrophage activation remains unknown, and no selective ligand is currently available	(Civil Ürkmez et al., 2024; Vay et al., 2020)
HCN2	Constitutively expressed in primary microglia; expression not altered by LPS	Best-supported functional isoform in cultured microglia: siRNA-mediated knockdown or ZD7288 depolarizes microglia, reduces proliferation, enhances survival of resting cells, and attenuates both M1-like and M2-like polarization while increasing IL-4-driven phagocytosis	Strongly cAMP-regulated (as is HCN4). HCN2 is a plausible—but unconfirmed—effector of Gi-coupled α2A signaling in microglia. Because HCN inhibition attenuates M2-like polarization while enhancing IL-4-associated phagocytosis, its net effect on reparative function remains uncertain	(Biel et al., 2009; Civil Ürkmez et al., 2024; Vay et al., 2020)
HCN3	Detectable in resting primary microglia; regulation by inflammatory stimuli not characterized	No isoform-specific function established in myeloid cells	Unknown; its relatively weak cAMP sensitivity may make a major contribution to α2A-dependent signaling less likely, but this has not been tested in myeloid cells	(Vay et al., 2020)
HCN4	Detectable in resting primary microglia; principal pacemaker isoform of the sinoatrial node	No isoform-specific myeloid function established	No myeloid therapeutic function has been established. Its principal relevance is the cardiovascular liability associated with systemic non-selective HCN inhibition (e.g., ivabradine), owing to its established role in sinoatrial pacemaking	(Biel et al., 2002; Roubille and Tardif, 2013; Vay et al., 2020)

Evidence concerning HCN isoforms in myeloid cells is derived from a small number of *in vitro* studies using an immortalized murine macrophage cell line and primary rodent microglia. The LPS-induced increase in HCN1 was also demonstrated in endothelial cells, which are not myeloid cells and are included only as supporting evidence of inflammation-associated HCN1 induction. HCN isoform expression does not by itself establish an isoform-specific function. ZD7288 is a non-isoform-selective HCN channel blocker with potential off-target effects, whereas isoform-specific knockdown evidence is currently available principally for HCN2. No study has established isoform-specific HCN functions in human microglia or macrophages *in vivo* or in autism spectrum disorder. Accordingly, the disease and therapeutic implications presented here are hypotheses derived from the available experimental evidence rather than established therapeutic effects. The terms M1-like and M2-like describe the experimental polarization conditions used in the cited studies and do not represent fixed or mutually exclusive myeloid-cell states. α2A, α2A-adrenoceptor; cAMP, cyclic adenosine monophosphate; G_i, inhibitory G protein; HCN, hyperpolarization-activated cyclic nucleotide-gated; IL, interleukin; LPS, lipopolysaccharide; M1/M2, M1-like/M2-like polarization states; siRNA, small interfering RNA.

These distinctions have divergent therapeutic implications (Figure 2B). HCN1-directed or activation-state-dependent blockade might dampen macrophage activation with comparatively little cardiac liability; HCN2-directed blockade would be predicted to dampen microglial reactivity broadly, but at the cost of impairing reparative polarization and debris clearance, which is undesirable in neurodevelopmental and post-injury contexts; and indirect modulation through α2A-mediated lowering of cAMP, as achieved with guanfacine, may offer a graded alternative that does not require isoform-selective ligands and might better preserve the capacity for reparative responses, although this has not been tested. Several caveats constrain these inferences: the isoform-resolved data in myeloid cells derive from a small number of *in vitro* studies in cell lines and primary rodent cultures; no study has profiled HCN isoform expression in microglia from autistic brain tissue or in human microglia *in vivo*; and isoform-selective pharmacological tools for HCN1–HCN3 remain unavailable, so the assignment of function to individual isoforms currently rests largely on expression data and siRNA-mediated knockdown.

It is useful to state the resulting logic as an explicit hierarchy because the four isoforms are not alternative targets of equivalent standing. At the top of the hierarchy lies the upstream node: Gi-coupled α2A-adrenoceptors, which are selectively upregulated on activated microglia and which lower cAMP without discriminating among isoforms. Since HCN2 is strongly regulated by cAMP—a property it shares with HCN4—and is the only isoform whose knockdown reproduces the phenotype of pharmacological blockade in microglia, cAMP lowering would be expected to act predominantly, though not exclusively, through HCN2. HCN1 occupies a second node that is state-dependent rather than constitutive: it is recruited by inflammatory stimulation, so its contribution should scale with the degree of activation and should be negligible in resting microglia. HCN3 occupies a third position in which expression is documented but function is not, and where its comparatively low cAMP sensitivity argues against a major role in α2A-mediated effects. No isoform-specific myeloid function has yet been established for HCN4; based on current evidence, it constrains the therapeutic approach from outside rather than participating in the microglial mechanism since its role as the sinoatrial pacemaker channel sets the cardiovascular ceiling on any systemically administered HCN blocker. Considered this way, the isoform data do not so much identify a single molecular target as they explain why an upstream, receptor-mediated, and graded intervention is pharmacologically more attractive than direct channel blockade: guanfacine reduces the drive to whichever cAMP-sensitive isoforms are expressed at a moment, in proportion to the activation state of the cell, without imposing the all-or-none loss of function produced by channel blockade and without engaging HCN4 in the heart. Whether receptor-mediated cAMP modulation does in fact preserve reparative polarization has not been tested and remains unknown.

The limits of this evidence warrant a fuller statement than a single caveat allows because the isoform assignments above carry a considerable inferential load. The macrophage data derive from RAW 264.7 cells, an immortalized murine line whose channel complement need not reflect that of primary human macrophages; the microglial data derive from primary rodent culture, in which microglia rapidly lose their *in vivo* transcriptional signature; siRNA-mediated knockdown and ZD7288 are each incompletely selective, the latter having documented off-target actions on T-type calcium and other currents at commonly used concentrations; and no study has examined HCN isoforms in microglia acutely isolated from brain, in human induced pluripotent stem cell-derived microglia, or in postmortem tissue from individuals with ASD. Three lines of work would place the framework on a firmer ground. Isoform-resolved single-cell or single-nucleus transcriptomic and proteomic profiling of microglia from ASD and control postmortem cortex would establish whether the isoform repertoire is itself altered in the condition related to this review. Conditional, myeloid-restricted deletion of Hcn1 or Hcn2 *in vivo* would separate the two isoforms without reliance on non-selective blockers. Measurement of microglial cAMP with genetically encoded sensors during α2A stimulation would test whether guanfacine actually achieves, in myeloid cells and at clinically attainable concentrations, the cAMP reduction that the proposed mechanism requires. We have therefore framed the isoform account as a compilation of the available evidence together with the therapeutic inferences it licenses, rather than as an established isoform-specific mechanism.

One hypothesis is that HCN currents help control the microglial “ramified” resting state; a depolarizing I_h current might promote motility and surveillance by resting microglia. Upon activation, changes in cyclic nucleotide levels (e.g., increased cAMP in certain contexts) could further modulate microglial HCN activity, potentially promoting a transition to an amoeboid, migratory phenotype. Microglia substantially alter their adrenergic receptor expression when activated, switching from predominantly β2-adrenergic receptor expression in the resting to upregulated α2A-adrenergic receptor expression in the LPS-activated state (Gyoneva and Traynelis, 2013). These shifts could intersect with HCN channel regulation.

In summary, accumulating data indicate that macrophages and microglia not only express HCN channels but utilize them as part of the cellular response to inflammation, a phenomenon particularly relevant to ASD, where microglial dysregulation is well documented (Morgan et al., 2010; Suzuki et al., 2013; Tetreault et al., 2012; Vargas et al., 2005). Considered together with the neuronal evidence above, HCN channels therefore constitute a single molecular node whose isoform composition and functional consequences differ by cell type—dampening dendritic integration in pyramidal neurons while supporting activation and polarization in microglia and macrophages—and this dual localization defines the two reciprocal guanfacine–HCN axes examined in the following two sections: a neuronal axis governing prefrontal excitatory/inhibitory balance, and a myeloid axis governing neuroinflammation.

## 4 The guanfacine–HCN–neuron axis: cAMP signaling, PFC function, and cortical excitatory/inhibitory balance in ASD

Elevated cAMP signaling disrupts persistent firing in prefrontal networks (Harnett et al., 2015; Kettenmann et al., 2011). Stress-induced catecholamine release increases intracellular cAMP and weakens working memory (Salter and Stevens, 2017; Tremblay et al., 2010). α2A receptor activation counteracts this effect by reducing cAMP production (Heneka et al., 2014; Prinz et al., 2021), explaining the protective role of guanfacine during stress exposure (Hains et al., 2015; Nimmerjahn et al., 2005). The pro-cognitive effects of guanfacine is fundamentally tied to its regulation of the cAMP–HCN pathway in PFC neurons (Arnsten, 2020; Wang et al., 2007). α2A-adrenoceptors localize to dendritic spines of PFC pyramidal cells, in proximity to ion channels that govern synaptic efficacy. The behavioral consequences of α2A stimulation depend critically on where these receptors are located. Postsynaptic α2A-adrenoceptors on the spines and dendrites of PFC pyramidal neurons mediate the pro-cognitive actions of guanfacine, whereas presynaptic α2A autoreceptors on locus coeruleus neurons and their terminals reduce noradrenergic outflow and account for the sedative, bradycardic, and hypotensive effects that limit dosing (Aoki et al., 1994; Arnsten, 2020; Arnsten et al., 1988). Guanfacine’s comparatively low potency at these autoreceptors, relative to that of clonidine, is the pharmacological basis for its wider therapeutic window, and it explains why cognitive benefit can be obtained at doses that produce little sedation (Arnsten et al., 1988). Under arousal or stress, elevated norepinephrine engages low-affinity β1/β2-adrenergic receptors, increasing cAMP in PFC neurons, leading to the opening of HCN and related K^+^ channels, which diminish PFC firing (Arnsten, 2020; Arnsten et al., 2023). This cascade explains stress-induced impairment of working memory, as cAMP–protein kinase A (PKA) activation and HCN channel opening functionally disconnect PFC recurrent networks (Arnsten, 2020; Ramos et al., 2003). Guanfacine counters this process by stimulating postsynaptic α2A-receptors on PFC spines, which are Gi-coupled and inhibit adenylyl cyclase, thereby reducing cAMP (Arnsten, 2020). The reduction in cAMP closes HCN and related Ca^2+^-dependent K^+^ channels on the spine, strengthening synaptic inputs and enhancing persistent firing of PFC neurons (Wang et al., 2007).

Wang et al. (2007) provided seminal evidence for this model, showing that α2A-agonists, such as guanfacine, improve working memory in monkeys by inhibiting cAMP signaling and HCN channel-mediated currents in dorsolateral PFC neurons. Iontophoretic application of guanfacine increased delay-related firing of PFC “memory cells,” an effect reversed by co-application of cAMP analogs (Arnsten, 2020). Conversely, HCN channel blockade with specific antagonists mimicked guanfacine’s strengthening effect of PFC neuronal firing, while activating HCN channels (via increased cAMP or β-stimulation) impaired firing and working memory (Arnsten, 2020). These findings have been replicated across species. In aged rats and monkeys, elevated PFC cAMP and HCN activity contribute to cognitive deficits alleviated by guanfacine. In aged monkeys guanfacine improved working memory, an effect abolished when cAMP analog was infused concurrently (Arnsten, 2020; Ramos et al., 2006). Chronic guanfacine treatment can also induce neuroprotective adaptations in PFC circuits. Daily guanfacine administration during 3 weeks of restraint stress prevented dendritic spine loss in the prelimbic PFC and preserved working memory relative to that in stressed controls (Hains and Arnsten, 2008; Ramos et al., 2003). These animals exhibited PFC synaptic density and cognitive function comparable to those of unstressed animals, an effect attributed to sustained inhibition of stress-induced cAMP–PKA–HCN signaling (Hains and Arnsten, 2008; Hains et al., 2015).

Several of these studies are causal rather than merely correlative, which is important because much of the clinical literature is observational. Iontophoretic guanfacine increases delay-related firing of PFC memory cells, and this effect is abolished by co-application of a cAMP analog; conversely, HCN channel blockade reproduces the effect and cAMP or β-adrenergic stimulation reverses it, and shRNA-mediated knockdown of HCN1 in the rat PFC improves working memory (Wang et al., 2007). Chronic guanfacine administration during restraint stress prevents dendritic spine loss with parallel preservation of behavior, and the pro-cognitive effect of α2A stimulation in aged animals is abolished by concurrent cAMP infusion (Hains et al., 2015; Ramos et al., 2006). What is not yet available is a causal test within an autism model: no study has manipulated α2A-adrenoceptors or HCN channels in a genetic or environmental ASD model and measured autism-relevant behavioral outcomes, so the causal chain established in stress and aging paradigms remains an inference when applied to ASD. These findings align with clinical observations that guanfacine can protect or restore PFC-related cognitive function under stress, fatigue, or hypoxia (Fesharaki-Zadeh et al., 2023b). Case reports have shown that patients with hypoxic brain injury or encephalitis receiving guanfacine regained attentional capacities; possibly through re-engagement of PFC networks compromised by neuromodulatory imbalances (Millichap, 2011).

In summary, the guanfacine enhances PFC function by suppressing cAMP signaling and closing HCN channels, thereby maintaining the excitatory recurrent connectivity required for working memory (Arnsten, 2020). This mechanism—initially elucidated in animal models—has been reproduced in human ADHD therapeutics and is being explored in other conditions characterized by PFC hypoactivity, including ASD (Arnsten, 2020; Arnsten et al., 2023).

The same axis has a second, circuit-level consequence that must be considered alongside its effect on individual pyramidal neurons: because HCN channels are differentially deployed in excitatory and inhibitory elements of the cortical microcircuit, α2A stimulation alters excitatory/inhibitory (E/I) balance rather than excitability uniformly. A central pathophysiological model of ASD posits a shift in the ratio of excitation to inhibition (E/I) within cortical circuits, arising from reduced GABAergic inhibitory tone, altered interneuron development or migration, or excessive glutamatergic drive (Nelson and Valakh, 2015; Rubenstein and Merzenich, 2003; Yizhar et al., 2011). In the PFC, parvalbumin (PV)-expressing fast-spiking interneurons provide the perisomatic inhibition that sets gamma-band synchrony and the signal-to-noise ratio of pyramidal ensembles, and their dysfunction is repeatedly implicated in genetic models of autism (Ferguson and Gao, 2018). Optogenetic restoration of E/I balance in the medial PFC by increasing PV interneuron excitability rescues social deficits in *Cntnap2*-deficient mice, establishing PFC E/I balance as a causally relevant and pharmacologically tractable node rather than an epiphenomenon (Selimbeyoglu et al., 2017).

Three considerations link the guanfacine–HCN axis to this E/I framework. First, HCN channels are expressed not only in pyramidal dendrites but also in GABAergic interneurons, where fast-spiking cells carry a prominent I_h that is concentrated in the axon and shapes action potential initiation, conduction reliability, and sustained high-frequency firing (Aponte et al., 2006; Roth and Hu, 2020). Because I_h in these interneurons supports rather than dampens excitability—the opposite of its role in pyramidal spines—cAMP-dependent modulation of HCN channels acts on the two limbs of the cortical microcircuit with opposite sign, so that the net consequence of α2A stimulation is a change in E/I ratio rather than a uniform change in excitability. Second, α2A-adrenoceptor stimulation increases the signal-to-noise ratio of PFC delay-related firing: guanfacine strengthens spatially tuned inputs to memory cells while responses to non-preferred directions are relatively suppressed, a profile functionally equivalent to sharpened inhibitory sculpting of the pyramidal ensemble (Arnsten, 2020; Wang et al., 2007). Third, PV interneurons are metabolically demanding and particularly vulnerable to oxidative and cytokine stress, so the inhibitory limb of the circuit is a plausible target of the neuroinflammation described below; by lowering microglial cytokine output, guanfacine may indirectly protect interneuronal function (Arnsten et al., 2023). Together, these mechanisms predict that α2A agonism normalizes PFC E/I balance through a cell-type-specific action on HCN channels, complemented by immune protection of interneurons (Figure 2A).

Because the two limbs of the microcircuit are modulated with opposite sign, the predicted sequence of events is worth setting out explicitly. First, tonic or phasic noradrenergic release from locus coeruleus terminals engages postsynaptic α2A-adrenoceptors on the spines of layer III PFC pyramidal neurons. Second, Gi-coupled inhibition of adenylyl cyclase lowers local cAMP and reduces probability of HCN channel opening at the spine neck, thereby increasing the electrotonic efficacy of recurrent excitatory inputs onto preferred-direction memory cells. Third, in parallel, the axonal I_h of PV fast-spiking interneurons, which sustains reliable high-frequency spiking, is subject to the same cAMP-dependent gating; if α2A-mediated cAMP lowering also reduced interneuronal I_h, perisomatic inhibition of non-preferred pyramidal ensembles would be attenuated rather than enhanced, which would oppose the pyramidal effect. Fourth, the outcome that is actually observed—increased delay-related firing for preferred directions together with relatively suppressed responses to non-preferred directions—therefore implies one of three things: that spine HCN channels are the dominant target at therapeutic receptor occupancy; that interneuronal I_h is comparatively insensitive to Gi-coupled cAMP lowering because axonal HCN channels in these cells operate near maximal activation at resting membrane potential; or that α2A-adrenoceptor expression is itself biased toward pyramidal spines relative to PV interneurons. Fifth, superimposed on this acute electrophysiological arithmetic is a slower, immune-dependent term: because chronic cytokine exposure and oxidative stress preferentially compromise metabolically demanding PV interneurons, a reduction in microglial cytokine output would shift the E/I ratio toward inhibition over days to weeks, in the same direction as, but by a mechanism distinct from, the acute channel effect. Distinguishing among these alternatives is the crux of the model because they make divergent predictions as to whether the acute and chronic actions of guanfacine on E/I balance are additive, redundant, or partially opposed.

This formulation should be read as a testable extension of the framework rather than an established mechanism. Direct measurements of I_h in identified PFC interneurons following α2A stimulation have not been reported, the sign and magnitude of interneuronal HCN modulation by Gi-coupled α2A signaling are unknown, and no study has measured E/I balance—electrophysiologically, with magnetic resonance spectroscopy or electroencephalographic gamma-band measures—in an autism model or in autistic individuals treated with guanfacine. Cell-type-specific electrophysiology in interneurons and E/I-sensitive outcome measures in clinical trials are the experiments that would arbitrate this prediction.

Four experiments would arbitrate between these possibilities, and we set them out because a framework of this kind is of little value unless it is falsifiable. First, whole-cell recordings from identified PV- and somatostatin-expressing interneurons in PFC slices, performed with and without an α2A agonist and with I_h isolated pharmacologically, would establish the sign and magnitude of interneuronal HCN modulation; this is the single most consequential unknown in the account given above. Second, cell-type-resolved quantification of α2A-adrenoceptor and HCN1/HCN2 protein in pyramidal spines versus interneuron axons by immuno-electron microscopy or proximity labeling would test the expression-bias explanation. Third, conditional deletion of HCN1 or HCN2 restricted to PV interneurons, combined with systemic guanfacine administration, would determine whether the inhibitory limb is required for, opposes, or is simply irrelevant to the behavioral effect. Fourth, in an established ASD model—maternal immune activation or Cntnap2 deletion, in both of which prefrontal E/I abnormalities and altered interneuron function have been described—chronic guanfacine could be evaluated against simultaneous electrophysiological, gamma-band electroencephalographic, and behavioral endpoints, which would test the acute and the immune-dependent terms of the model in the same animals. Until such data become available, the E/I formulation should be regarded as a mechanistic hypothesis that reorganizes existing observations rather than as a conclusion supported by direct evidence. We retain it because it generates the falsifiable predictions listed above, accommodates the otherwise puzzling observation that a single receptor mechanism improves signal-to-noise rather than simply increasing or decreasing cortical activity, and identifies interneuronal HCN modulation as the specific measurement the field currently lacks.

## 5 The guanfacine–HCN–neuroinflammation axis: noradrenergic modulation of microglia and macrophages, and the evidence for guanfacine and its limits

Peripheral immune activation alters hippocampal and prefrontal plasticity (Dantzer et al., 2008; McKim et al., 2018). Increased IL-6 correlates with reduced executive performance (Perry et al., 2010; Ransohoff, 2016). Chronic inflammation promotes recruitment of peripheral monocytes to the brain (Cunningham, 2013; Teeling and Perry, 2009). These immune changes are associated with cognitive decline (Arnsten et al., 2012; Ramos and Arnsten, 2007). Neuroinflammation—characterized by microglial activation and peripheral macrophage infiltration—is well-established in a wide spectrum of conditions, ranging from delirium and postoperative cognitive dysfunction to neurodegenerative diseases (Alam et al., 2018; Tan et al., 2023), and is increasingly recognized in ASD (Masi et al., 2015; Morgan et al., 2010; Suzuki et al., 2013; Vargas et al., 2005). Inflammatory cytokines impair synaptic plasticity and affect neural circuits involved in memory and attention (Cibelli et al., 2010; Lynch, 2010). Elevating IL-1β in the brain (via peripheral immune challenge or surgery) can disrupt long-term potentiation and induce memory deficits—effects prevented by IL-1 receptor blockage (Cibelli et al., 2010). Cibelli et al. (2010) demonstrated that peripheral surgery triggers an IL-1β-mediated inflammatory response in the hippocampus, leading to postoperative cognitive dysfunction in rodents, whereas IL-1β knockout or antagonism abolished this impairment. Aging is accompanied by a pro-inflammatory central milieu (elevated IL-6 and TNF-α) tightly linked to synaptic and cognitive decline (Nikas, 2013). Age-related increases in microglial inflammatory markers correlate with impaired long-term potentiation and reduced spatial memory, suggesting that inflammatory cytokines may be primary contributors to age-related cognitive deficits (Lynch, 2010). These insights and the parallel literature documenting microglial activation and cytokine elevations in postmortem and immune-profiling studies in autism, highlight why anti-inflammatory strategies are being explored across neurodevelopmental and cognitive disorders.

Noradrenergic signaling is an endogenous modulator of neuroinflammation. NE from locus coeruleus fibers interacts with microglial adrenergic receptors and influences activation state. Microglia highly express β2-adrenergic receptors under basal conditions (Gyoneva and Traynelis, 2013; Mishima et al., 2019), and NE acting on β2 tends to suppress microglial pro-inflammatory activities while promoting a ramified surveillance morphology (Damo et al., 2023). Upon immune challenge or stress, microglia upregulate α2A-adrenergic receptor expression, which mediate NE’s effects in the activated state. NE bidirectionally modulates microglial motility through different adrenoceptors: β2-receptors in resting microglia cause process retraction (reduced surveillance branching), and α2A-receptors in LPS-activated microglia also induce process retraction and curtailed migration toward ATP. NE signaling—depending on receptor engagement—can “calm” microglial movement and dampen inflammatory responses. NE blocked the microglial process extension normally triggered by ATP in brain slices, and β-receptor antagonism prolonged microglial responses to ATP, indicating that endogenous NE puts the brakes on microglial reactivity (Gyoneva and Traynelis, 2013).

Beyond the central nervous system, NE and epinephrine modulate peripheral macrophage polarization and cytokine output (Freire et al., 2022; Qiao et al., 2018). β-adrenergic stimulation shifts macrophages toward an anti-inflammatory (M2-like) phenotype, increasing IL-10 and decreasing TNF-α, whereas α2-adrenergic signaling can exert similar effects under certain conditions (Li et al., 2025; Qiao et al., 2018). Activation of α2-adrenoceptors in alveolar macrophages drove the M1-to-M2 polarization switch that reduced inflammatory injury in a rodent model of acute lung inflammation (Li et al., 2025). Li et al. (2025) showed α2-agonist treatment after kidney ischemia-reperfusion promoted alveolar macrophages to adopt an anti-inflammatory profile (increased Arg-1, IL-10 expression) and mitigated lung damage, an effect abolished by α2 signaling blockade. Adrenergic drugs can thus act as immune modulators, altering the trajectory of inflammation in ways that may protect neural circuits.

Stress, which elevates catecholamine levels, often induces an initial anti-inflammatory effect via β2-adrenergic action (sometimes followed by compensatory pro-inflammation) (Johnson et al., 2013; Vida et al., 2024). Chronic stress increases neuroinflammatory signaling and drives recruitment of peripheral monocytes to the brain, contributing to anxiety and cognitive disturbance (Tan et al., 2023). Targeting adrenergic pathways could help break the cycle of stress and inflammation. Propranolol, a non-selective β-blocker, has been explored to prevent trauma-induced neuroinflammation and anxiety, though with mixed clinical results. α2-agonists, such as dexmedetomidine (Dex) and clonidine, are used clinically to attenuate sympathetic outflow and have demonstrated anti-inflammatory benefits in perioperative and critical care settings. Dex reduces the release of high-mobility group box-1 in sepsis models (Arnsten et al., 2023) and inhibits LPS-induced cytokine production by activating macrophage peroxisome proliferator-activated receptor gamma (PPAR-γ) (Han et al., 2022). In a study by Fujimoto et al. (2021), Dex-induced suppression of TNF-α and IL-6 in LPS-stimulated macrophage cultures was reversed by an α2-antagonist or a PPAR-γ antagonist, confirming that Dex acts through α2-ARs to engage the PPAR-γ pathway and blunt inflammatory responses. Sedative doses of Dex in surgical patients have been linked to lower circulating IL-6 and C-reactive protein postoperatively, with improved recovery of cognitive function (Chen et al., 2021; Qi et al., 2022). A randomized trial in 86 patients undergoing intestinal surgery found that perioperative Dex significantly downregulated levels of IL-6, IL-8, and TNF-α while upregulating IL-10 levels compared with those in controls (Chen et al., 2021).

Taken together, modulating macrophage/microglia activity via adrenergic receptors can influence inflammation-driven cognitive outcomes (Table 4). α2A agonists stand out as candidates to achieve this, given their dual actions on neurons (enhancing PFC function) and immune cells (limiting pro-inflammatory activation). This strategy is directly relevant to autism, where both PFC circuit and microglial mechanisms are implicated.

**TABLE 4:** Preclinical and clinical evidence relevant to α2-adrenoceptor-mediated immunomodulation.

Agent	Study type	Model/setting	Key anti-inflammatory findings	References
Guanfacine	Preclinical (rodent)	Chronic restraint stress	Preserved PFC dendritic spines and working memory; microglial engulfment of synapses was not measured	(Hains et al., 2015)
Norepinephrine/α2A agonism (guanfacine not tested)	*In vitro*	Cultured microglia; ATP- and chemokine-evoked motility	Reduced motility and chemotactic response via α2A-AR; α2A-AR upregulated on activated microglia	(Gyoneva and Traynelis, 2013)
Guanfacine + N-acetylcysteine	Clinical (open-label)	Long COVID cognitive syndrome	Improved executive function over 8 weeks; no inflammatory markers measured and NAC co-administered, so the guanfacine-specific contribution cannot be isolated	(Fesharaki-Zadeh et al., 2023b)
Dexmedetomidine	Preclinical (*in vitro*)	LPS-stimulated macrophages	Suppressed TNF-α, IL-6 via α2-AR → PPAR-γ activation	(Fujimoto et al., 2021)
Dexmedetomidine	Preclinical (*in vitro* and mouse)	LPS-induced inflammation	Attenuated inflammatory cytokine responses via macrophage IL-10 expression following α7 nAChR activation	(Han et al., 2022)
Dexmedetomidine	Clinical RCT	Intestinal surgery patients	Lower IL-6 and CRP postoperatively	(Chen et al., 2021)
α2 agonist	Preclinical (rat)	Kidney ischemia-reperfusion → lung	Alveolar macrophage M1→M2 polarization; mitigated lung injury	(Qiao et al., 2018)

Evidence obtained with dexmedetomidine, norepinephrine, and other α2-adrenoceptor agonists is presented as class-level evidence for α2-adrenoceptor-mediated immunomodulation and should not be interpreted as evidence of a guanfacine-specific anti-inflammatory action. Direct immunological evidence for guanfacine remains limited. The microglial motility findings were obtained using norepinephrine and α2A-adrenoceptor-selective pharmacology rather than guanfacine. Furthermore, neither the chronic restraint-stress study nor the available clinical studies measured inflammatory markers during guanfacine treatment. In the Long COVID series, NAC was co-administered; therefore, the contribution of guanfacine could not be isolated. α2A-AR, α2A-adrenoceptor; α7 nAChR, α7 nicotinic acetylcholine receptor; ATP, adenosine triphosphate; CRP, C-reactive protein; IL, interleukin; LPS, lipopolysaccharide; M1/M2, M1-like/M2-like macrophage polarization states; NAC, N-acetylcysteine; PFC, prefrontal cortex; PPAR-γ, peroxisome proliferator-activated receptor gamma; RCT, randomized controlled trial; TNF-α, tumor necrosis factor alpha.

Against this background of class-level adrenergic immunomodulation, the evidence bearing specifically on guanfacine, as distinct from the α2 agonist class as a whole, can now be examined and its limits stated. Beyond its neuronal effects, guanfacine may exert immunomodulatory effects in the central and peripheral immune systems, although specific evidence remains limited and much of the supporting literature concerns norepinephrine or other α2-adrenoceptor agonists (Tan et al., 2023). As an α2A-agonist, guanfacine can deactivate microglia and macrophages through the mechanisms described above. Preclinical studies have shown that guanfacine and related α2-agonists reduce microglial activation in various models of neuroinflammation. In cultured microglia, norepinephrine acting through α2A-adrenoceptors diminished motility and blunted the response to ATP or chemotactic stimuli; guanfacine itself was not administered in that study (Gyoneva and Traynelis, 2013). By extension from receptor pharmacology, an α2A-selective agonist, such as guanfacine, might maintain microglia in a more surveillant and less reactive state during inflammatory challenge, but this is an inference rather than a demonstrated action of the drug. In a rodent model of chronic psychological stress that causes PFC spine loss, chronic guanfacine administration preserved dendritic spines and working memory performance (Hains et al., 2015). Microglial engulfment of synapses was not measured in that study; the possibility that microglia mediate part of this protection is an extrapolation from the separate literature on microglia-dependent synaptic pruning (Paolicelli et al., 2011; Schafer et al., 2012), and not a direct observation. Such protective effects have been attributed in part to direct actions on microglia: α2A-receptors are induced on activated microglia, and their stimulation has been proposed to “deactivate” microglia, limiting excessive phagocytosis and inflammatory cytokine release; this proposal, however, rests on receptor-expression data and on studies employing norepinephrine or other α2 agonists rather than guanfacine (Arnsten, 2020; Gyoneva and Traynelis, 2013). Guanfacine might therefore attenuate stress-induced synaptic pruning by microglia—a possibility that has not been tested directly—complementing its role in strengthening synapses from the neuronal side—a mechanism with clear relevance to ASD, where aberrant microglial pruning is a leading hypothesis for altered cortical connectivity (Arnsten, 2020; Arnsten et al., 2023; Hong et al., 2016; Paolicelli et al., 2011; Parkhurst et al., 2013; Schafer et al., 2012; Stevens et al., 2007).

In peripheral immune models, α2-agonists show similar anti-inflammatory effects. In an LPS-stimulated mouse model, Dex reduced macrophage TNF-α, IL-6, and IL-1β while increasing IL-10 via α7 nicotinic acetylcholine receptor activation (Han et al., 2022). Guanfacine shows greater α2A-selectivity and possibly exerts its effects with fewer off-target actions. Theα2-adrenoceptor signaling in LPS-stimulated human monocyte-derived macrophages has been described as shifting these cells toward an anti-inflammatory transcriptional profile, with increased IL-10 and reduced IL-1β secretion; this account derives from a narrative review of adrenergic regulation of myeloid cells rather than from a primary experiment in which guanfacine itself was applied to these cells (Nayak et al., 2024). At the molecular level, guanfacine’s anti-inflammatory effects in immune cells likely involve intracellular pathways, such as PKA, mitogen-activated protein kinase, and nuclear factor kappa B. α2A signaling can inhibit adenylate cyclase (lowering cAMP), which is linked to decreased production of inflammatory mediators in macrophages. Additionally, α2-AR activation in macrophages increases production of 15d-PGJ2, a downstream product of cyclooxygenase metabolism that activates PPAR-γ and induces an anti-inflammatory gene program (Fujimoto et al., 2021). Guanfacine might leverage this same PPAR-γ-mediated mechanism as observed with Dex, acting on the same receptor and cell types.

Clinical experience with guanfacine in inflammation-associated cognitive syndromes is limited to small, uncontrolled series in which no inflammatory marker was measured; these reports therefore describe cognitive outcomes and cannot serve as *in vivo* evidence of an anti-inflammatory action. In a pilot open-label study of patients with “Long COVID” cognitive syndrome, the combination of guanfacine (1 mg at bedtime, increased to 2 mg after 1 month) and N-acetylcysteine (600 mg daily) improved working memory, concentration, and executive function in 8 of 12 patients, with 2 patients discontinuing the intervention due to hypotension or dizziness; because N-acetylcysteine was co-administered and no inflammatory or cytokine markers were assessed, this series can attribute neither the cognitive benefit nor any immunological effect to guanfacine specifically (Fesharaki-Zadeh et al., 2023b). The authors proposed that guanfacine’s dual role in enhancing PFC networks and reducing neuroinflammation may alleviate persistent “brain fog” after COVID-19. Although uncontrolled, this aligns with prior evidence that guanfacine can ameliorate cognitive deficits in conditions associated with inflammation and cytokine excess, such as post-concussive syndrome and chemobrain. A trial in patients with mild TBI found that a single dose of guanfacine altered blood-oxygen-level-dependent responses in the PFC and modestly improved working memory, suggesting that guanfacine might normalize aberrant catecholamine and immune-related signaling post-injury (McAllister et al., 2011). In a case of autoimmune encephalitis with severe inattention and neglect, guanfacine was associated with clinically apparent gains in sustained attention and reduced spatial neglect on bedside examination; because this was a single case report, no standardized quantitative cognitive endpoints, effect sizes, or confidence intervals were available (Singh-Curry et al., 2011). Deficits in arousal and sustained attention, likely driven by inflammatory damage to subcortical attention pathways, improved during treatment, which is consistent with—but does not establish—an anti-inflammatory or neuromodulatory therapeutic effect. While these observations are not autism-specific, they provide preliminary evidence for cognitive benefit in inflammation-associated conditions but do not demonstrate immune engagement by guanfacine.

Overall, collective findings from cell culture, animal models, and clinical reports indicate that α2-adrenoceptor agonism attenuates inflammatory responses in microglia and macrophages, and thereby suggest—rather than demonstrate—that guanfacine would act similarly through α2A-adrenoceptors on these cells. At the class level, this evidence includes decreased pro-inflammatory cytokine production, limited immune cell activation and motility, and promotion of a return to homeostatic conditions (Fesharaki-Zadeh et al., 2023a; Fujimoto et al., 2021). If these actions extend to guanfacine, they would complement its direct neuronal effects, together fostering an environment conducive to PFC circuit function. This putative dual profile of simultaneously strengthening cortical networks and damping neuroinflammation (Figure 1 and Table 5) distinguishes guanfacine from other cognitive enhancers and has spurred interest in repurposing it for conditions such as ASD, delirium, and post-COVID cognitive syndrome, where PFC hypoactivity and inflammation co-occur (Arnsten et al., 2023).

**TABLE 5:** Comparison of established neuronal actions of guanfacine and proposed immune-cell actions inferred from α2-adrenoceptor agonism.

Aspect	Neuronal actions (PFC)	Proposed immune-cell actions (microglia/macrophages; inferred from α2-agonist class evidence)	References
Target receptor	Postsynaptic α2A-AR on PFC pyramidal dendritic spines	Upregulated α2A-AR expression on activated microglia and macrophages	(Arnsten, 2020; Gyoneva and Traynelis, 2013; Wang et al., 2007)
G-protein coupling	Gi-coupled inhibition of adenylyl cyclase →↓cAMP	Gi-coupled inhibition of adenylyl cyclase →↓cAMP in immune cells	(Arnsten, 2020; Han et al., 2022)
Downstream effector	HCN channel closure on dendritic spines → strengthened synaptic input	Suppression of NF-κB, MAPK; activation of PPAR-γ pathway	(Fujimoto et al., 2021; Han et al., 2022; Wang et al., 2007)
Cellular outcome	Enhanced persistent (delay-related) neuronal firing; preserved dendritic spines	Reduced motility and cytokine release; shift toward M2-like phenotype	(Paolicelli et al., 2011; Qiao et al., 2018; Ramos et al., 2006)
Functional outcome	Improved working memory, attention, and executive function	Decreased neuroinflammation; ↓TNF-α, IL-1β, IL-6; ↑IL-10	(Arnsten, 2020; Han et al., 2022; Vida et al., 2024)
Clinical relevance	ADHD, ASD, PTSD, TBI, stress-related PFC dysfunction	Delirium, post-COVID brain fog, perioperative cognitive dysfunction, neurodevelopmental neuroinflammation	(Arnsten et al., 2023; Fesharaki-Zadeh et al., 2023b; Rajendran and Paolicelli, 2018)

The neuronal actions shown in this table are supported by direct experimental evidence with guanfacine. In contrast, the immune-cell actions represent a proposed framework inferred primarily from studies of norepinephrine, dexmedetomidine, and other α2-adrenoceptor agonists conducted in different experimental systems. These immune pathways have not been demonstrated as a unified mechanism or directly confirmed with guanfacine in microglia or macrophages. The arrows indicate proposed directional relationships and should not be interpreted as evidence that all signaling steps occur sequentially within the same cell type. The clinical conditions listed in the immune-cell column represent potential translational contexts rather than established indications or evidence of guanfacine-specific immunomodulatory efficacy. α2A-AR, α2A-adrenoceptor; ADHD, attention-deficit/hyperactivity disorder; ASD, autism spectrum disorder; cAMP, cyclic adenosine monophosphate; G_i, inhibitory G protein; HCN, hyperpolarization-activated cyclic nucleotide-gated; IL, interleukin; MAPK, mitogen-activated protein kinase; NF-κB, nuclear factor kappa B; PFC, prefrontal cortex; PPAR-γ, peroxisome proliferator-activated receptor gamma; PTSD, post-traumatic stress disorder; TBI, traumatic brain injury; TNF-α, tumor necrosis factor alpha.

## 6 Translational and clinical implications for ASD and related conditions

Anti-inflammatory interventions improve cognitive outcomes in experimental models (Arnsten, 2009; Ramos and Arnsten, 2007). Noradrenergic drugs reduce microglial activation and cytokine production (Damo et al., 2023; Gyoneva and Traynelis, 2013). Clinical studies suggest that α2 agonists may reduce delirium risk (Birnbaum et al., 2004; Yang et al., 2013). These observations support therapeutic targeting of neuroimmune interactions (Rajendran and Paolicelli, 2018), including in ASD, where chronic low-grade neuroinflammation co-occurs with executive and attention difficulties.

Understanding the relationships among guanfacine, HCN channels, and macrophages provides valuable insights for developing treatments across neurology, psychiatry, and neurodevelopment. From a neuroscience perspective, guanfacine’s ability to tune HCN channel activity in PFC neurons offers a targeted approach to enhance cognitive function. Unlike stimulants that broadly increase catecholamine levels, guanfacine fine-tunes endogenous signaling, restoring optimal network firing by closing improperly opened HCN channels (e.g., due to stress or aging-related cAMP dysregulation) (Ramos et al., 2003). This explains its efficacy in PFC disorders, such as ADHD, and suggests potential in other conditions with frontal executive dysfunction, including ASD, vascular cognitive impairment, and chemotherapy-related cognitive changes. Small trials in cognitively impaired older adults have hinted at benefits in frontal-lobe tasks (Arnsten, 2020), and neuroimaging studies in monkeys show that guanfacine increases blood flow in the PFC during working memory performance, consistent with enhanced neural activity.

From an immunology perspective, guanfacine may represent a candidate immunomodulatory strategy with potential relevance to both central and peripheral inflammatory states. However, direct evidence specific to guanfacine’s immunological effects remains limited, underscoring the need for targeted investigation. Its use as a sedative adjunct in critical care (e.g., Dex) already capitalizes on its delirium-sparing, immunomodulatory effects (Arnsten et al., 2023). The possibility that guanfacine deactivates microglia and macrophages without completely suppressing immune function is attractive: α2-adrenoceptor agonism is thought to bias these cells toward a neuroprotective, lower-inflammatory phenotype rather than to cause broad immunosuppression, although this has not been demonstrated for guanfacine directly (Arnsten, 2020). This could benefit the treatment of conditions such as TBI, where secondary neuroinflammation worsens outcomes; preliminary clinical experience in patients with TBI has been encouraging, with case series reporting improved working memory and reduced impulsivity in patients with moderate TBI treated with guanfacine, alongside anecdotal reductions in neuroinflammatory markers (Fesharaki-Zadeh et al., 2025).

Several lines of reasoning suggest that this dual mechanism is particularly attractive for autism. First, atypical PFC connectivity and executive dysfunction are core features in many individuals with autism, and guanfacine’s mechanism of strengthening recurrent PFC firing addresses this directly. Second, chronic microglial activation and elevated cytokines are observed in a substantial subset of individuals with autism, and the proposed capacity of α2A-adrenoceptor agonism to deactivate microglia without suppressing immune function would, if confirmed for guanfacine, offer a more graded therapeutic approach than that provided by broad immunosuppression. Third, the high rates of co-occurring ADHD-like symptoms, irritability, sensory hyperreactivity, and stress reactivity in autism, all of which involve PFC and noradrenergic mechanisms, fit well with guanfacine’s established clinical profile. Adequately powered, autism-specific randomized trials—ideally incorporating candidate stratification measures such as peripheral cytokine profiles or microglial neuroimaging—would be a logical next step. We note, however, that no cytokine or imaging measure has yet been validated as a diagnostic or predictive biomarker in autism, so these approaches remain exploratory (Masi et al., 2017).

Age and sex are underexplored moderators of guanfacine response and deserve explicit consideration. Autism-specific efficacy data are restricted largely to children and adolescents aged 6–17 years (Politte et al., 2018; Scahill et al., 2015). To our knowledge, no study has directly compared treatment effects across age groups or examined age-by-treatment or sex-by-treatment interactions in ASD. Guanfacine is not, however, without adult evidence: it showed positive effects in a phase 3 randomized controlled trial in adults with ADHD (Iwanami et al., 2020), and *post-hoc* analyses of adult ADHD trial data have examined efficacy in subgroups defined by age and sex (Naya et al., 2021). It, however, exhibited negative effects in a randomized trial conducted in adults aged 75 years and older (Barcelos et al., 2018), but because the diagnosis, outcome measures, and population all differed from those of the pediatric autism trials, this is better regarded as a failure of generalization to a different older cohort than as a demonstration of an age effect. The hypothesis that α2A–HCN-directed treatment is most effective before age-related dysregulation of cAMP signaling and loss of dendritic spines become structurally entrenched (Arnsten, 2020) is consistent with preclinical evidence of age-dependent shifts in PFC cAMP–HCN signaling in aged rats and monkeys (Ramos et al., 2006; Wang et al., 2011), but it is not established by the clinical data reviewed here.

Sex effects remain largely unexplored. Clinical trials of guanfacine in autism and ADHD have enrolled predominantly male participants, sex-disaggregated efficacy and tolerability analyses are rarely reported. Similarly, preclinical investigations of the α2A–HCN axis have relied on male animals almost exclusively. This gap is not trivial: microglial transcriptional and functional phenotypes are sexually dimorphic across development and in adulthood (Hanamsagar and Bilbo, 2016; Villa et al., 2018). Moreover., autism is diagnosed more frequently in males and may be underrecognized in females, and cardiovascular tolerability of α2 agonists may differ by sex and body mass. Addressing these gaps requires age-stratified and sex-stratified analyses in clinical research, alongside the deliberate inclusion of female animals in mechanistic studies. Such efforts are critical to advancing a more comprehensive understanding of this axis.

Two further considerations underscore that the absence of sex-stratified data is more than a formal omission. Mechanistically, the noradrenergic system is itself sexually dimorphic: locus coeruleus neurons in female rodents have more extensive dendritic arbors and greater afferent integration, corticotropin-releasing factor signaling within the locus coeruleus is more readily engaged in females, and autoreceptor-mediated feedback differs between sexes (Bangasser et al., 2016). Because guanfacine’s therapeutic window is set precisely by the balance between its postsynaptic prefrontal action and its autoreceptor-mediated sedative and hypotensive actions, that window need not be the same in males and females, and the optimal dose may therefore differ even where efficacy does not. Immunologically, microglial density, transcriptional state, and cytokine responsiveness differ by sex and by developmental stage, and the timing of microglial colonization and of synaptic pruning is itself sex-dependent, so an intervention whose putative benefit is partly immune-mediated could show sex-dependent efficacy and, more importantly, a sex-dependent optimal window for intervention (Hanamsagar and Bilbo, 2016; Villa et al., 2018). Clinically, the practical consequence is that an evidence base drawn from cohorts in which male participants substantially outnumber female participants, with no reported sex-by-treatment interaction analyses, can exclude neither a smaller nor a larger effect in females. Therefore, future trials should pre-specify sex as a moderator rather than treat it as a nuisance covariate, and sex-disaggregated efficacy and adverse-event data including cardiovascular measures must be reported. Additionally, preclinical studies of this axis should include both sexes by design. Until this is done, the generalizability of the framework across sex should be treated as unknown rather than assumed.

Integrated therapeutics targeting both neural and immune dysfunction may be especially valuable for syndromes, such as ASD, “brain fog” in long COVID, postoperative delirium, and age-related cognitive decline. In these scenarios, excessive inflammation (cytokines, activated microglia) coincides with impaired PFC network connectivity. Guanfacine’s dual action— strengthening PFC circuits via HCN channel closure and reducing inflammation—directly targets this pathological duet (Fesharaki-Zadeh et al., 2023b). A recent review by Arnsten et al. (2023) underscored its relevance in clinical trials evaluating guanfacine therapy in delirium and long COVID cognitive impairment. An NIH-funded trial is underway testing guanfacine for post-acute sequelae of COVID, and two phase 2 trials are examining guanfacine for delirium in critically-ill patients (Fesharaki-Zadeh et al., 2023b). Outcomes of these trials may expand guanfacine’s indications and validate a therapeutic paradigm addressing neuroimmune contributors to cognitive dysfunction, including in neurodevelopmental contexts.

## 7 Limitations

This is a narrative, mechanism-focused review rather than a systematic review or meta-analysis, and thus its scope and conclusions are constrained. We did not perform a pre-registered systematic search, and study selection was guided by relevance to the proposed neuronal–immune framework; some relevant work may therefore have been omitted. The autism-specific evidence base for guanfacine remains limited: most randomized trials focus on co-occurring ADHD-like symptoms and short-term outcomes, with comparatively little data on core social-communication features, long-term effects, or cognitive endpoints linked directly to PFC function. Direct mechanistic evidence linking HCN channel dysfunction to ASD remains preliminary, and we are unaware of randomized trials that have measured neuroinflammatory biomarkers in individuals with autism receiving guanfacine. Much of the immunological evidence is derived from *in vitro* studies, rodent models, or perioperative use of Dex—a related but pharmacologically distinct α2-agonist—and extrapolation to chronic outpatient guanfacine use in individuals with autism should be made cautiously. Effect sizes from clinical trials on guanfacine are heterogeneous and often modest, available trials are typically underpowered to detect subgroup effects, and confidence intervals around reported benefits are wide. Guanfacine’s sedative side effects (fatigue, hypotension) can limit dosing, particularly in fragile patients and younger children (Iwanami et al., 2020). Cognitive benefits also appear most pronounced in individuals with baseline PFC dysregulation; in healthy individuals or under optimal conditions, guanfacine may have minimal effects or even cause slight impairments due to ceiling effects on PFC firing (Arnsten, 2020). Patient selection, including identification of individuals with autism who are most likely to benefit, is therefore critical. Finally, although guanfacine influences immune activity, it is not an anti-inflammatory drug; its effects are modulatory and may not suffice in severe cytokine storms or fulminant inflammation and therefore, combination therapy may be warranted (e.g., pairing guanfacine with an antioxidant or anti-cytokine agent, as was piloted with N-acetylcysteine in long COVID) (Fesharaki-Zadeh et al., 2023b). The evidence base is also unbalanced with respect to age and sex. The autism-specific efficacy data is derived primarily from school-aged children and adolescents (Handen et al., 2008; Scahill et al., 2006, 2015; Politte et al., 2018), and no study has examined age-by-treatment or sex-by-treatment interactions in ASD, and sex-disaggregated analyses of efficacy or tolerability are almost never reported in either clinical or preclinical studies of this axis. In addition, isoform-resolved data on HCN channels in microglia and macrophages is from a small number of *in vitro* studies, and no isoform-selective pharmacological tools are available for HCN1–HCN3. Readers and clinicians should interpret the synthesis as a translational framework that motivates further research rather than as evidence supporting clinical recommendation for autism.

## 8 Conclusion

Integrative approaches targeting both neural and immune mechanisms are increasingly recognized as effective strategies. Modulation of neuromodulatory signaling may restore network stability. Future therapies may combine synaptic and immunological interventions, with potential benefits across neuropsychiatric and neurodevelopmental conditions, including ASD. Decades after its introduction, guanfacine has evolved from an antihypertensive drug into an agent for cognitive enhancement and neuropsychiatric therapy. This review highlights how guanfacine’s engagement with α2A-adrenergic receptors inhibits cAMP signaling, yielding two major benefits: (1) closure of HCN channels on PFC neurons to strengthen synaptic connectivity and cognitive function, and (2) deactivation of microglia and macrophages to reduce inflammatory stress on the brain. These dual actions confer guanfacine a unique profile among cognitive enhancers, directly addressing the “hyperpolarized” and “inflamed” PFC characteristic of many cognitive and neurodevelopmental disorders, including ASD. The extensive literature surveyed in this review underscores a unifying principle: optimizing the PFC environment at both synaptic and immune levels is critical for restoring cognition. Further research is needed to translate these findings into targeted therapies; the role of HCN channels in immune cells is still in early stages, and the autism-specific application of this framework requires direct empirical testing. Nevertheless, the available evidence supports the concept that therapies bridging neuronal and immune mechanisms hold promise for autism and related conditions. Continued interdisciplinary research, including studies of HCN channel modulators, α2-agonist derivatives optimized for neuroinflammatory conditions, and biomarkers of HCN channel activity and microglial state, will be essential. The intricate interplay among guanfacine, HCN channels, and macrophages may herald a new direction in neuropsychopharmacology, in which cognitive enhancement and anti-inflammatory neuroprotection are pursued simultaneously to treat and prevent ASD and related cognitive disorders.
